# Phosphoproteomic Profiling of Selenate-Treated Alzheimer's Disease Model Cells

**DOI:** 10.1371/journal.pone.0113307

**Published:** 2014-12-08

**Authors:** Ping Chen, Lixiang Wang, Yong Wang, Shuiming Li, Liming Shen, Qiong Liu, Jiazuan Ni

**Affiliations:** 1 College of Life Sciences, Shenzhen Key Laboratory of Microbial Genetic Engineering, Shenzhen University, Shenzhen, 518060, China; 2 Shenzhen Key Laboratory of Marine Biotechnology and Ecology, Shenzhen University, Shenzhen, 518060, China; 3 Key Laboratory of Optoelectronic Devices and Systems of Ministry of Education and Guangdong Province, College of Optoelectronic Engineering, Shenzhen University, Shenzhen, 518060, China; McGill University Department of Neurology and Neurosurgery, Canada

## Abstract

The reversible phosphorylation of proteins regulates most biological processes, while abnormal phosphorylation is a cause or consequence of many diseases including Alzheimer's disease (AD). One of the hallmarks of AD is the formation of neurofibrillary tangles (NFTs), which is composed of hyperphosphorylated tau proteins. Sodium selenate has been recently found to reduce tau hyperphosphorylation and NFTs formation, and to improve spatial learning and motor performance in AD mice. In the current study, the phosphoproteomics of N2aSW cells treated with selenate were investigated. To avoid missing low-abundance phosphoproteins, both the total proteins of cells and the phosphor-enriched proteins were extracted and subjected to the two-dimensional gel electrophoresis with Pro-Q diamond staining and then LC-MS/MS analysis. A total of 65 proteins were altered in phosphorylation level, of which 39 were up-regulated and 26 were down-regulated. All identified phosphoproteins were bioinformatically annotated according to their physiochemical features, subcellular location, and biological function. Most of these significantly changed phosphoproteins are involved in crucial neural processes such as protesome activity, oxidative stress, cysteine and methionine metabolism, and energy metabolism. Furthermore, decreases were found in homocysteine, phosphor-tau and amyloid β upon selenate treatment. Our results suggest that selenate may intervene in the pathological process of AD by altering the phosphorylation of some key proteins involved in oxidative stress, energy metabolism and protein degradation, thus play important roles in maintaining redox homeostasis, generating ATP, and clearing misfolded proteins and aggregates. The present paper provides some new clues to the mechanism of selenate in AD prevention.

## Introduction

Protein phosphorylation is one of the most ubiquitous post-translational modifications involved in regulating a majority of biological processes. It is required for proper protein folding, and functions as a signal for further protein modifications such as ubiquitination. Phosphorylation may alter protein subcellular localization, induce conformational changes, alter catalytic activity, and modify protein-protein interactions. Protein phosphorylation is regulated by a highly dynamic network of kinases and phosphatases. At least one-third of eukaryotic proteins are phosphorylated [1], among them only a subset are modified by any given stimulus. Abnormal phosphorylation is a cause or consequence of many human diseases including Alzheimer's disease (AD) [2], [3].

AD is an age related neurodegenerative disease affecting 36 million people worldwide and is the most common form of dementia. Clinically, AD is characterized by impaired memory, deterioration of intelligence and emotion, formation of neuritic amyloid plaques and neurofibrillary tangles (NFTs), neuron loss and subsequent behavior deficits. Extracellular amyloid plaques (also called senile plaques, SPs) and intracellular NFTs in the brain regions of neocortex, entorhinal cortex, and hippocampus are two principal histopathological hallmarks of AD patients. SPs are mainly composed of misfolded amyloid-β peptide (Aβ), which is formed by the proteolytic processing of amyloid precursor protein (APP). NFTs consist of hyperphosphrylated microtubule associated protein tau and occur in the neuronal cell body and dystrophic neurites. Abnormal phosphorylation of tau decreases its binding affinity with microtubules and causes its dissociation from microtubules, resulting in cytotoxicity and aggregating into NFT. Besides tau, aberrant phosphorylation of several other proteins such as neurofilaments, β-catenin and microtubule-associated protein 1B have also been found to associate with AD pathogenesis, confirming that altered phosphorylation is a common event during AD progression [3]. Thus, phosphorylation analysis and phosphorylated-protein identification become crucial in studying the pathogenesis of AD.

Selenium is essential for proper brain function [4]. Low dietary selenium is reported to be associated with poor cognitive function [5]–[8]. Some selenium compounds have been found to be able to reduce AD pathology in cell culture and animal models. Seleno-L-methionine (Se-Met) could protect cell against Aβ-induced oxidative stress and toxicity in primarily cultured neurons [9]. It was also found to ameliorate cognitive decline, reduce tau hyperphosphorylation and reverse synaptic deficit in the triple transgenic mouse model of AD [10]. Sodium selenite inhibited amyloid production by decreasing γ-secretase activity and mitigating cognitive impairment in a streptozotocin-induced rodent model of AD [11]. Sodium selenate could specifically activate protein phosphatase 2A (PP2A), dephosphorylate tau and reverse memory deficits in several AD models [12], [13]. Due to its low toxicity and potential effect in AD treatment, sodium selenate becomes a compound that attracts many researchers to study its mechanism behind the biological function.

In the current study, we used a phosphoproteomic approach to identify the altered phosphoproteins in AD model cells N2aSW, treated with or without sodium selenate. Total proteins extracted from cell lysates and the phosphoproteins enriched from total proteins were analyzed by two dimensional gel electrophoresis (2DE) plus Pro-Q diamond staining followed by LC-MS/MS detection, where Pro-Q Diamond is a fluorescent phosphorsensor capable of sensitive detection of phosphoserine-, phosphothreonine-, and phosphotyrosine-containing proteins. Western blotting and ELISA assays were further used to investigate the impact of selenate on AD pathology related proteins.

## Materials and Methods

### Materials and Reagents

N2aSW cells were kindly provided by Professor Huaxi Xu and Yunwu Zhang in Xiamen University. DMEM, Opti-MEM and fetal bovine serum (FBS) were purchased from Hyclone (Logan, UT, USA); penicillin and streptomycin were from Meck (Whitehouse Station, USA). Sodium selenate was purchased from Sigma-Aldrich (Shanghai, China). CCK-8 and RIPA lysis were obtained from Beyotime Biotech (Shanghai, China). Halt phosphatase inhibitor and Pierce Phosphoprotein Enrichment Kit were purchased from Pierce (USA). Pro-Q Diamond phosphoprotein gel stain kit, SYPRO Ruby gel stain kit, immobilized pH gradient (IPG) strips and related chemicals used in two-dimensional gel electrophoresis (2DE) were obtained from GE Healthcare (Guangzhou, China). Primary antibodies against β-actin, Tau, Tau (phosphor-S404), Tau (phosphor-T231), Tau (phosphor-S422), Tau (phosphor-S396), BACE1, APP and horse-radish peroxidase (HRP)-conjugated mouse or rabbit IgG were purchased from Abcam Biochemicals (Cambridge, UK). The polyvinylidene fluoride (PVDF) membrane was obtained from Millipore (Madison, USA). Enhanced chemiluminescence for the immune complexes in Western blot experiments was visualized using a Pierce ECL detection kit (Thermo Fisher Scientific Inc., Rockford, USA). All other reagents were obtained from GE Healthcare, unless stated otherwise. Homocysteine (Hcy) assay kit was the product of AUSA Pharmed Ltd (China). Mouse Aβ ELISA kit was purchased from Airan Tech. Corp. (Beijing, P. R. China).

### Cell culture and viability assay

N2aSW cell line was derived from the N2a neuroblastoma cell stably expressing APPsw, a Swedish mutant of APP protein [14]. Those cells were cultured in DMEM/Opti-MEM supplemented with 5% fetal bovine serum, 0.2 mg/mL G418, 100 units/mL streptomycin and 100 units/mL penicillin, and maintained in a humidified atmosphere with 5% CO_2_ at 37°C. When the cells had reached 80% confluence, they were harvested and plated for subsequent passage or drug treatment.

Cell viability was detected by the CCK-8 assay. Briefly, N2aSW cells (5×10^3^ per well) exposed to sodium selenate at a set of concentrations (0.00001, 0.0001, 0.001, 0.01, 0.1, 1, 10 mmol/L in FBS-free medium) were grown in 96-well-plates for 24 h. 10% CCK-8 reagent was added to each well and the cells were incubated at 37°C for another 2 h. Cells without sodium selenate treatment were used as the control. The absorbance at 450 nm was measured with a reference wavelength of 650 nm by SpecTRA MAX 190 microplate reader (Molecular Devices, Sunnyvale, CA, USA). All experiments were performed in triplicates.

### Protein extraction

N2aSW cells were cultured with or without 0.01 mmol/L sodium selenate for 24 h. The untreated cells served as the control. After cell-harvest and wash with PBS three times, those cells were suspended in a lysis buffer containing 7 mol/L urea, 2 mol/L thiourea, 4% CHAPS, 120 mmol/L DTT, 2% IPG buffer (pH 3-10), 30 mmol/L Tris-HCl, 2 mmol/L protease inhibitor (PMSF) and Halt phosphatase inhibitor, and incubated at 4°C for 30 min. Subsequently, the samples were ultrasonicated for 1 min in a mode of 1 s on and 3 s off, using a Fisher 550 Sonic Dismembrator (Pittsburgh, PA, USA) at 15% power. Cell suspensions were then centrifuged at 13,000 g to remove unsoluble debris. Protein concentrations were determined by the Bradford assay. Phosphoproteins were enriched by the Pierce Phosphoprotein Enrichment Kit (Pierce Biotechnology), according to the protocol.

### Two dimensional gel electrophoresis and image acquisition

Proteins were separated in the first dimension by isoelectric focusing (IEF). Equal amounts of samples (100 µg) were loaded onto a 13-cm Immobiline Drystrip gel (pH 4–7 nonlinear). Rehydration process was performed at 30 V for 12 h. Hydration and IEF was performed in the following conditions: 100 V for 2 h, 200 V for 1 h, 500 V for 1 h, 1000 V for 1 h, 1000–3000 V by gradient in 1 h, 3000–8000 V by gradient in 3 h, and finally 8000 V for 7 h.

Following IEF, the immobiline strips were equilibrated with a reducing equilibration buffer (6 mol/L urea, 75 mmol/L Tris-HCl, pH 8.8, 30% glycerol, 2% SDS, 1% DTT) for 15 min at room temperature. The strips were subsequently incubated in the same buffer but containing 4.5% iodoacetamide (IAA) instand of DTT. Proteins on the equilibrated strips were further separated on 12% SDS-PAGE gels at 15°C by the Ettan DALTsix Electrophoresis System (GE Healthcare) under 15 mA for 30 min and then 40 mA to the end. The 2-DE separated proteins were visualized with Pro-Q Diamond for phosphoproteins and with SYPRO Ruby for total proteins. Gel images were taken by the Typhoon laser scanner and then analyzed using the Image Master 2D Platinum software. Data were obtained in triplicate and normalized to remove differences in protein loading and staining. The differential protein spots (more than 1.5-fold and P<0.05) were picked up for further analysis.

### In-gel tryptic digestion

After running the preparative gel using identical 2-DE conditions as above, these gels were stained with silver nitrate solution. The differential protein spots identified were manually matched to the Pro-Q Diomand stained gel. Spots were excised from the silver stained gel and destained with 50% acetonitrile and 25 mmol/L NH_4_HCO_3_. Following destaining, the gel-pieces were digested with 10 µL of 1% trypsin in 25 mmol/L NH_4_HCO_3_ at 37°C overnight. The resulting peptides were subjected to LC-MS/MS analysis.

### Mass spectrometry and database searching

Protein identification was carried out by an AB SCIEX MALDI-TOF/TOF 5800 mass spectrometry (Foster City, CA, USA). 1 µL of the peptide extract was dropped into the target which was pre-loaded with 1 µL of 10 mg/mL α-cyano-4-hydroxycinnamic acid (CHCA) dissolved in 0.1% TFA and 50% aceton, and dried at room temperature to crystallize before LC-MS/MS analysis. The spectra were externally calibrated. MASCOT was used for database searching against the UniProt databases (Matrix Science, UK). The search was carried out using the Mus musculus database, with a tolerance in mass measurement of 100 ppm in MS mode and 0.5 Da in MS/MS mode. Up to one missed cleavage per peptide was allowed and the fixed carbamidomethyl and phosphor-ST and phosphor-Y modification were taken into account. Information on protein MW and pI was also considered for protein identification based on the location of the protein spot in the 2-DE gel.

### Homocysteine analysis

To investigate the effect of sodium selenate on Hcy metabolism, the extracellular Hcy level was determined by Hcy assay kit. Briefly, cell culture media were collected and spinned to remove pellets. Protein concentrations in the culture media were determined using the Bradford assay. The following steps were performed according to the manufacturer's instruction.

### Western blotting

To study the effect of sodium selenate on AD pathology, Western blotting analyses of APP, BACE1, Tau, phosphor-Tau (S404), phosphor-Tau (T231), phosphor-Tau (S422) and phosphor-Tau (S396) were performed. Protein samples (40 µg) was separated on a 12% SDS polyacrylamide gel, followed by transferred onto polyvinylidene fluoride (PVDF) membranes. Membranes were blocked for 2 h in TBST buffer (137 mM NaCl, 2.7 mM KCl, 19 mM Tris, 0.05% Tween 20, pH 7.4) containing 5% nonfat milk, and incubated overnight at 4°C with the primary antibody. After three washes with TBST, the membranes were incubated for another 1 h at room tempreture with anti-rabbit or anti-mouse IgG HRP secondary antibody. Finally, the membranes were washed and visualized using chemiluminescence with an ECL kit (Pierce). β-actin was probed as an internal loading control.

### Enzyme-linked immunosorbent assay (ELISA)

The level of extracellular Aβ was measured by mouse Aβ ELISA kit according to the manufacturer's instructions. Aβ concentrations were interpolated from kit-specific standard curves generated in GraphPad Prism (GraphPad Software).

### Bioinformatics analysis

The differentially phosphorylated proteins were categorized using the PANTHER gene ontology database (http://pantherdb.org),and Database for Visualization and Integrated Discovery (DAVID) v6.7 (http://david.abcc.ncifcrf.gov/home.jps) were used to analyze pathways these proteins involved.

### Statistical Analysis

Data were analyzed using SPSS 13.0 statistical software (SPSS Inc., Chicago, Illinois, USA). Differences between groups were analyzed using one-way ANOVA and Student-Newman Keuls (SNK). The level of significance was set at *P*<0.05.

## Results

### Effect of sodium selenate on cell viability

**Figure 1:**
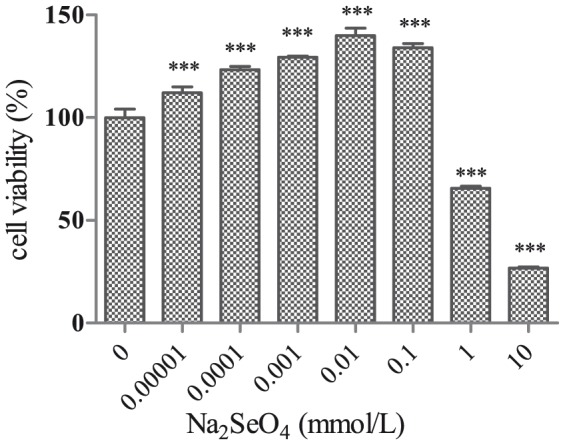
Dose-dependent effect of sodium selenate on the viability of N2aSW cells after 24 h treatment. ***, *P*<0.001.

Cell viability was evaluated by CCK-8 assay. As shown in Fig. 1, the growth of N2aSW cells was increased by sodium selenate dose-dependently. Only at a concentration higher than 0.01 mmol/L was it inhibited significantly by selenate. The optimum concentration of sodium selenate to increase cell viability was 0.01 mmol/L, which was selected as a dose for the treatment of selenate in the following experiments.

### Phosphoproteins altered in the total proteins of cells treated with selenate

**Figure 2:**
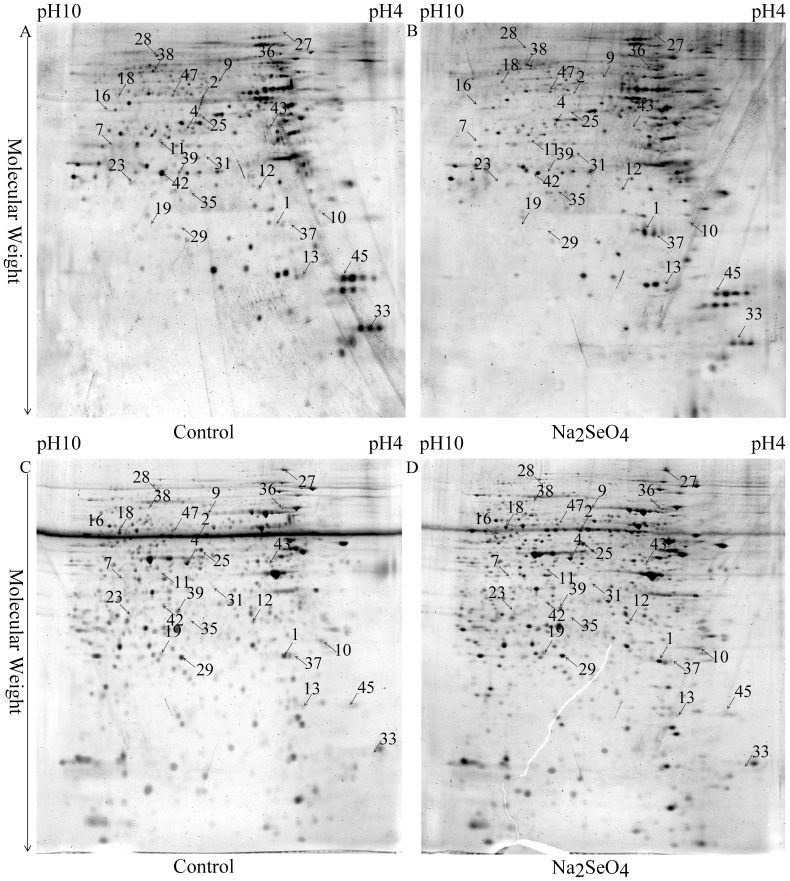
Representative 2-DE images of the total proteins extracted from the untreated (control) and selenate-treated N2aSW cells. Gels were stained with Pro-Q Diamond (A, B) and SYPRO ruby (C, D) fluorescent dyes, respectively. Arrows indicate the identified proteins whose phosphorylation levels were altered.

To study the effect of sodium selenate on protein phosphorylation in N2aSW cells, comparative phosphoproteomic analysis was implemented through a series of steps, including 2DE separation, image analysis, in-gel digestion, MS identification and database searching. Total proteins isolated from N2aSW cells treated with or without 0.01 mmol/L sodium selenate for 24 h were subjected to phosphoproteomic analysis. Typical 2-DE gel images are shown in Fig. 2. Protein spots with a fold-change ≥|1.5| and a *P*-value ≤0.05 were considered as significantly different phosphorylation in the cells with or without selenate-treatment. Twenty-five proteins were found differentially phosphorylated (Table 1). Ten of them showed increased phosphorylation, and fifteen identified phosphoproteins showed decreased phosphorylation in the selenate-treated cells. All identified phosphorylated peptides from the above proteins were presented in Table S1 in S1 File.

**Table 1:** Differentially phosphorylated proteins identified from the total proteins by 2-DE-LC-MS/MS between the selenate-treated and untreated N2aSW cells.

Spot No.^a^	Protein abbreviation	Protein name^b^	score	number of peptides matched (searched)	Mass (kDa)/pI^c^	Fold Exp^d^	Fold Ph^e^
1	UCHL1	Ubiquitin carboxyl-terminal hydrolase isozyme L1	3205	75	25165/5.14	0.96	6.91
2	TCPA	T-complex protein 1 subunit alpha	6436	137	60867/5.82	0.5	0.16
4	DNJA2	DnaJ homolog subfamily A member 2	638	28	46344/6.06	0.89	0.21
7	AATC	Aspartate aminotransferase, cytoplasmic	1984	85	46504/6.68	0.8	0.25
9	HSP7C	Heat shock cognate 71 kDa protein	3156	88	71055/5.37	0.68	3.89
10	ENOPH	Enolase-phosphatase E1	326	17	28696/4.79	0.79	3.58
11	SAHH	Adenosylhomocysteinase	5006	178	48170/6.08	0.79	0.28
12	CAZA2	F-actin-capping protein subunit alpha-2	2629	86	33118/5.57	0.89	0.31
13	LGUL	Lactoylglutathione lyase	1396	97	20967/5.24	1.29	3.18
16	RTCB	tRNA-splicing ligase RtcB homolog	3180	95	55727/6.77	2	3.05
18	TCPZ	T-complex protein 1 subunit zeta/T-complex protein 1 subunit zeta-2	11774	489	58424/6.63	1.05	2.99
19	RPIA	Ribose-5-phosphate isomerase	1549	40	32545/7.81	1.4	2.97
23	G3PDH	Glyceraldehyde-3-phosphate dehydrogenase	3233	147	36072/8.44	1.04	0.37
25	HNRH1	Heterogeneous nuclear ribonucleoprotein H	14878	548	49454/5.89	0.99	0.39
27	HYOU1	Hypoxia up-regulated protein 1	19347	745	111340/5.12	1.63	2.55
28	HS90B	Heat shock protein HSP 90-beta/Heat shock protein HSP 90-alpha	2881	126	83571/4.97	1	0.4
29	PRDX4	Peroxiredoxin-4	3979	116	31261/6.67	0.96	0.4
31	IDH3A	Isocitrate dehydrogenase [NAD] subunit alpha, mitochondrial	1805	90	40069/6.27	0.88	0.42
33	RLA1	60S acidic ribosomal protein P1	812	42	11582/4.28	0.64	0.43
36	NDUS1	NADH-ubiquinone oxidoreductase 75 kDa subunit, mitochondrial	4938	237	80752/5.51	0.22	0.47
37	RANG	Ran-specific GTPase-activating protein	3058	147	23753/5.15	0.81	2.11
38	GUAA	GMP synthase [glutamine-hydrolyzing]	4190	208	77416/6.29	1.2	0.48
39	MDHC	Malate dehydrogenase, cytoplasmic	2846	155	36659/6.16	0.75	0.48
43	KAP0	cAMP-dependent protein kinase type I-alpha regulatory subunit	1674	69	43443/5.27	0.79	0.49
47	SYSC	Serine—tRNA ligase, cytoplasmic	10417	519	58865/5.95	0.8	1.92

a Protein IDs were assigned manually.

b Protein names were identified by MS.

c Theoretical molecular weight and isoelectric point of the protein(s).

d The ratio in spot density from the Rubby stained gel of the selenate treated cells compared to the control (untreated cells).

e The ratio in spot density from the Pro-Q diomand stained gel of the selenate treated cells compared to the control (untreated cells).

### Phosphoproteins altered in the enriched proteins from the cells treated with selenate

**Figure 3:**
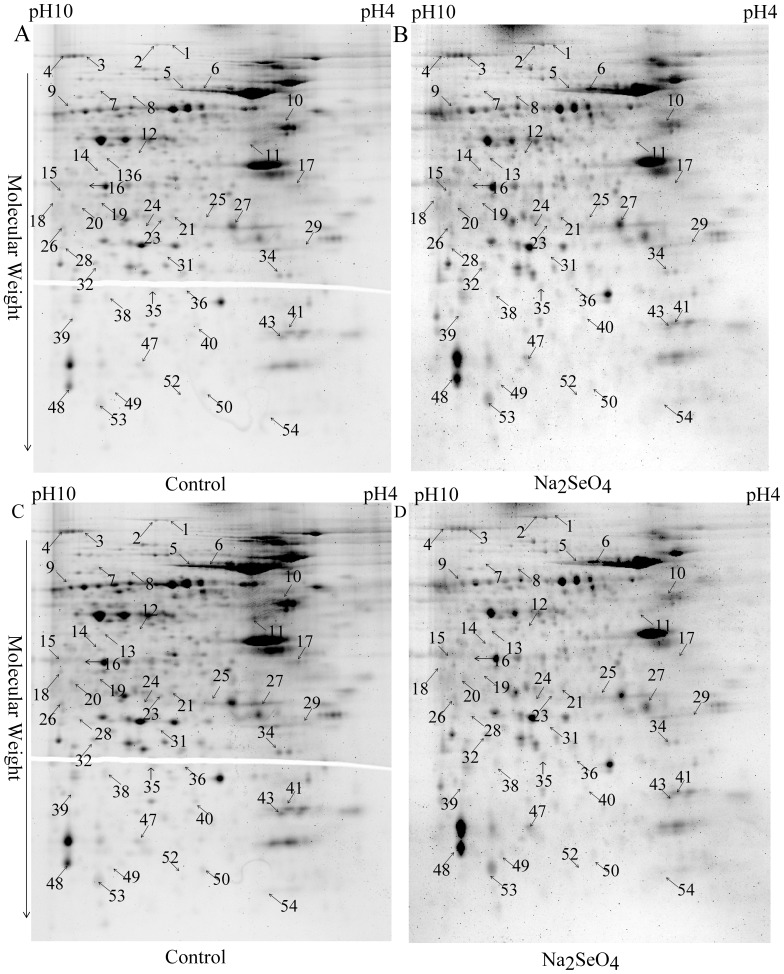
Representative 2-DE images of the phosphor-enriched proteins from the untreated (control) and selenate-treated N2aSW cells. Gels were stained with Pro-Q Diamond (A, B) and SYPRO ruby (C, D) fluorescent dyes, respectively. Arrows indicate the identified proteins whose phosphorylation levels were altered.

Generally, phosphoproteins with low abundance in the cells are difficult to be detected by proteomics due to the existence of highly abundant phosphoproteins. Thus, phosphoproteins were enriched from the total proteins for possible detection of low-abundance proteins, using the Pierce Phosphoprotein Enrichment Kit. The enriched proteins from the selenate-treated and nontreated N2aSW cells were then used for phosphoproteomic analyses. Representative 2DE gel images are shown in Fig. 3. A total of forty-five protein spots representing forty-one proteins were found to be phosphorylated differentially between the selenate-treated and untreated cells. Their identities were determined with LC-MS/MS and shown in Table 2. Thirty-seven phosphoprotein spots, corresponding to thirty-three phosphoproteins, showed significantly increased in the phosphorylated leveland meanwhile eight of them, whose phosphorylated levels were significantly decreased upon selenate treatment. All the identified phosphorylated peptides from the above proteins were presented in Table S2 in S1 File.

**Table 2:** Differentially phosphorylated proteins identified from the enriched proteins by 2-DE-LC-MS/MS between the selenate-treated and untreated N2aSW cells.

Spot No.^a^	Protein abbreviation	Protein name^b^	score	number of peptides matched (searched)	Mass (kDa)/pI^c^	Fold Exp^d^	Fold Ph^e^
1	VINC	Vinculin	8384	233	117215/5.77	2.16	2.5
2	VINC	Vinculin	16790	576	117215/5.77	2.83	2.39
3	EF2	Elongation factor 2	17323	578	96222/6.41	1.29	2.26
4	EF2	Elongation factor 2	19977	631	96222/6.41	1.6	2.04
5	SYK	Lysine—tRNA ligase	4855	243	68253/5.65	1.12	2.05
6	HSP7C	Heat shock cognate 71 kDa protein	7263	273	71055/5.37	2.28	3.12
7	TCPG	T-complex protein 1 subunit gamma	6154	252	61162/6.28	0.65	2.17
8	DPYL2	Dihydropyrimidinase-related protein 2	1499	48	62638/5.95	1.42	2.09
9	SYYC	Tyrosine—tRNA ligase, cytoplasmic	8843	379	59410/6.57	1.61	3.11
10	ATPB	ATP synthase subunit beta, mitochondrial	10434	301	56265/5.19	0.62	0.49
11	IF4A1	Eukaryotic initiation factor 4A-I	4468	139	46353/5.32	0.41	0.42
12	SEPT2	Septin-2	448	15	41727	2.31	2.81
13	HNRH1	Heterogeneous nuclear ribonucleoprotein H	1813	54	49454/5.89	2.55	2.22
14	HSP7C	Heat shock cognate 71 kDa protein	3055	90	71055/5.37	1.44	2.44
15	ALDOA	Fructose-bisphosphate aldolase A	930	31	39787	2.52	2.76
16	TWF2	Twinfilin-2	1359	33	39674/6.33	2.19	2.26
17	EEF1-delta	Elongation factor 1-delta	4947	133	31387	0.29	2.26
18	ALDR	Aldose reductase	1583	82	36052/6.71	1.6	0.41
19	ENOA	Alpha-enolase	2275	72	47453/6.37	1.68	2.16
20	PRPS1	Ribose-phosphate pyrophosphokinase 1	1540	54	35325	2.01	2.55
21	PRS6B	26S protease regulatory subunit 6B	1221	41	47493	2.29	3.55
23	5NT3B	7-methylguanosine phosphate-specific 5'-nucleotidase	1243	42	34688	2.58	3.31
24	PSA1	Proteasome subunit alpha type-1	1741	55	29813/6	0.17	2.37
25	KPYM	Pyruvate kinase isozymes M1/M2	1471	44	58378/7.18	2.67	2.98
26	MTAP	S-methyl-5'-thioadenosine phosphorylase	3747	156	31612/6.71	2.92	3.37
27	ACTBL	Beta-actin-like protein 2	2027	75	42319	1.87	2.05
28	CYBP	Calcyclin-binding protein	676	33	26608/7.63	1.82	2.24
29	SGTA	Small glutamine-rich tetratricopeptide repeat-containing protein alpha	1320	29	34529/4.99	2.07	0.46
31	PRDX6	Peroxiredoxin 6	873	33	24925/5.98	1.3	3.66
32	PSA2	Proteasome subunit alpha type-2	1369	53	26024/6.92	1.25	2.18
34	GDIR1	Rho GDP-dissociation inhibitor 1	2240	86	23450/5.12	1.69	0.23
35	EMC8	ER membrane protein complex subunit 8	658	20	23790/5.72	0.42	0.35
36	SERB	Phosphoserine phosphatase	853	30	25308	0.19	0.21
38	PSB2	Proteasome subunit beta type-2	941	28	23063	2.19	2.38
39	BLVRB	Flavin reductase (NADPH)	427	15	22297/6.49	2.23	2.98
40	ACTB	Actin, cytoplasmic 1	205	9	42052	1.69	4.35
41	PSB6	Proteasome subunit beta type-6	3466	119	25591/4.97	1.36	2.47
43	PRDX2	Peroxiredoxin-2	1628	65	21936/5.20	0.65	0.4
47	KPYM	Pyruvate kinase isozymes M1/M2	757	36	58378/7.18	3.64	3.26
48	NDKA	Nucleoside diphosphate kinase A	8479	396	17311/6.84	3.2	2.58
49	MGDP1	Magnesium-dependent phosphatase 1	1305	39	18628/6.29	4.33	4.05
50	MGN	Protein mago nashi homolog	790	36	17210/5.74	0.92	2.49
52	PFD5	Prefoldin subunit 5	852	28	17402	5.27	2.72
53	HINT1	Histidine triad nucleotide-binding protein 1	2916	119	13882/6.36	2.4	2.53
54	LEG1	Galectin-1	837	21	15198	5.32	2.57

a Protein IDs were assigned manually.

b Protein names were identified by MS.

c Theoretical molecular weight and isoelectrical point of the protein(s).

d The ratio in spot density from the Rubby stained gel of the selenate treated cells compared to the control (untreated cells).

e The ratio in spot density from the Pro-Q diomand stained gel of the selenate treated cells compared to the control (untreated cells).

### Gene ontology analysis of selenate-altered phosphoproteins

**Figure 4:**
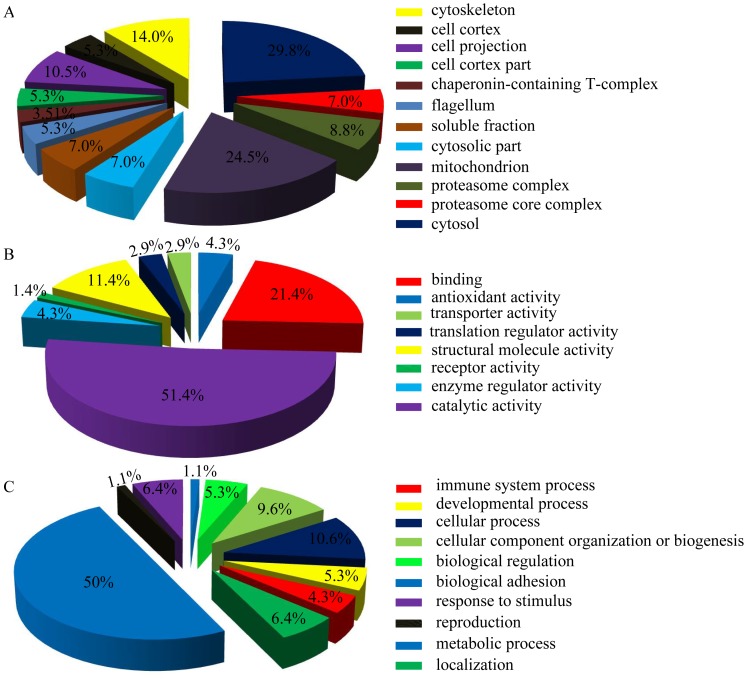
PANTHER gene ontology enrichment analysis of phosphoproteins altered in the N2aSW cells treated with sodium selenate. Enrichment analyses were performed in terms of cellular component (A), molecular function (B) and biological process (C).

In order to get the selenate-regulating phosphoproteome profile, all altered phosphoproteins identified in this paper were classified into cellular component, molecular function, and biological process based on the information in the Mouse Protein Reference Database (Fig. 4). According to the annotation of GO analysis, the altered phosphoproteins in terms of cellular component are widely distributed in various organelles, especially in cytosol and mitochondrion (accounting for 29.8% and 24.5% of the identified phosphoproteins, respectively) (Fig. 4A). In terms of molecular function, catalytic activity was the largest subgroup constituting 51.4% of the identified phosphoproteins, followed by binding ability (21.4%) and structural molecule activity (11.4%) (Fig. 4B). In terms of biological process, the altered phosphoproteins are mainly involved in metabolic process (50%), cellular process (10.6%), cellular component organization or biogenesis (9.6), response to stimulus (6.4%), localization (6.4%), and biological regulation (5.3%) (Fig. 4C).

### Major metabolic pathways in N2aSW cells affected by sodium selenate

For further analysis, the Database for Visualization and Integrated Discovery (DAVID) Bioinformatics Resources v6.7 was used to classify the altered phophoproteins into different signaling pathways. As shown in table 3, only 23 out of the 65 differential phosphoproteins were mapped onto the KEGG database and assigned to various metabolic pathways including proteasome, pentosephosphate pathway, cysteine and methionine metabolism, pyruvate metabolism, glycolysis/gluconeogenesis and purine metabolism.

**Table 3:** Canonical KEGG pathways associated with the altered phosphoproteins.

pathway	*p*-value	target molecules
proteasome	2.6×10^−4^	PRS6BPSA1, PSA2, PSB2, PSB6
pentose phosphate pathway	6.8×10^−4^	ALDOA, PRPS1, RPIA, TALDO
cysteine and methionine metabolism	1.4×10^−3^	ENOPH, MTAP, SAHH, AATC
pyruvate metabolism	2.6×10^−3^	ALDR, LGUL, MDHC, KPYM
glycolysis/gluconeogenesis	1.1×10^−2^	ALDOA, G3P, ENOA, KPYM
purine metabolism	9.0×10^−2^	GUAA, PRPS1, KPYM, NDKA

### AD pathological features inhibited by selenate-treatment

**Figure 5:**
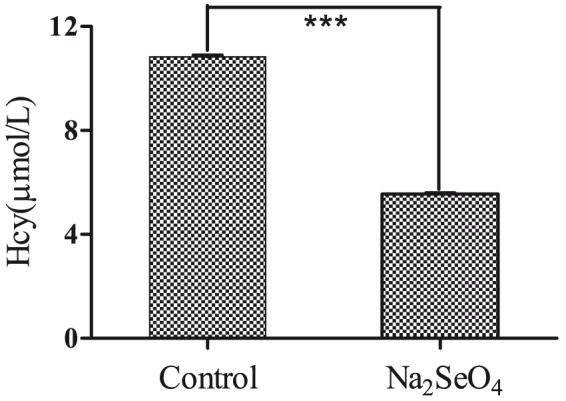
Supplementation of sodium selenate on extracellular Hcy level. Culture media of N2aSW cells were collected for the detection of Hcy level. ***, *P*<0.001.

**Figure 6:**
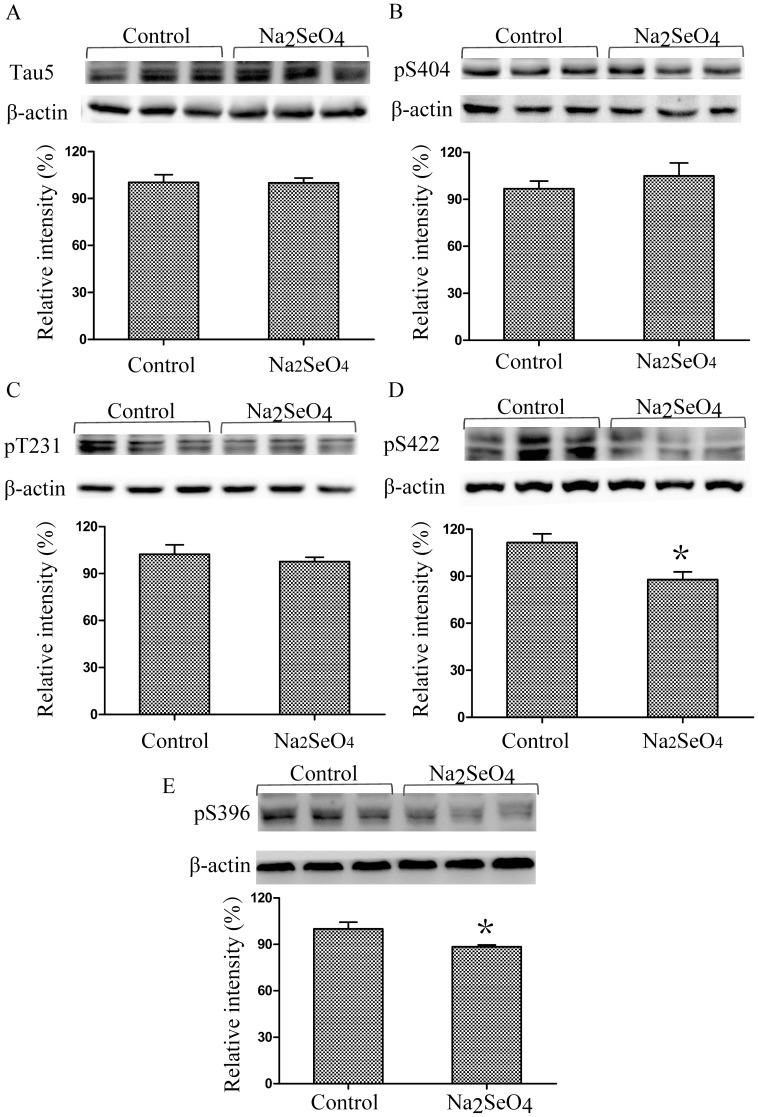
Effect of selenate on tau phosphorylation in N2aSW cells. Levels of total tau protein expression (A), phosphorylation of tau at pS404 (B) or at pT231 (C) were not altered in the cells treated with/without selenate. However, the phosphorylation level of tau at pS422 and pS396 were reduced by selenate-treatment (D). *, *P*<0.05.

**Figure 7:**
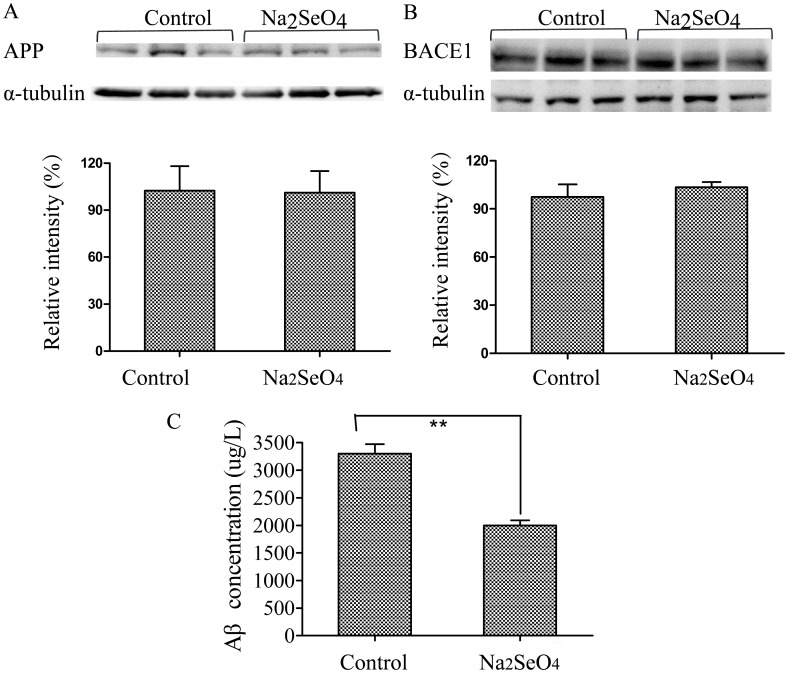
Sodium selenate regulated Aβ production in N2aSW culture medium. The expression levels of APP (A) and BACE1 (B) proteins did not change significantly between the selenate-treated and the control cells. While extracellular Aβ concentration was down-regulated when treated with selenate (C). **, *P<0.01*.

In order to investigate the effect of selenate on AD pathology of N2aSW cells, several Aβ- and tau-related proteins together with Hcy were selected for detection in this paper. As shown in Fig. 5, selenate (0.01 mmol/L) treatment of N2aSW cells resulted in a decline of Hcy level in the culture medium. Selenate treatment did not affect total tau protein expression level or phosphorylation levels of tau at pS404 and pT231, but reduced phosphorylation of tau at pS422 and pS396 (Fig. 6), which is consistent with previous reports in several other AD models [12], [13]. As shown in Fig. 7, sodium selenate could also decrease extracellular Aβ level, but it did not alter APP and BACE1 proteins expression in N2aSW cells, when compared with the untreated cells (the control).

## Discussion

The pathogenesis of AD is very complicated. One of them is the sequential cleavage of APP by β-secretase (BACE1) and γ-secretase to produce the Aβ peptide fragment that aggregates to damage cells directly or to increase tau hyperphosphorylation, resulting in cell apoptosis. A large body of evidence suggests that Aβ is central to the pathophysiology of AD and is likely to start this intractable neurodegenerative disorder. In N2aSW cells, i.e. N2a cells with stable transfection of the Swedish mutation of APP, it has been demonstrated that elevated Aβ levels resulted in the hyperphosphorylation of tau, leading to aberrant secondary structures and function loss in tau protein, and further the reduced abilities to bind to microtubules and to disturb their assemble [15]–[17]. Selenium is an important trace element for brain. The level of selenium is decreasing with the increase of age, and low level of selenium is closely related to the cognitive dysfunction and eventually AD [6], [7], [18], [19]. It has been reported that sodium selenate could improve spatial memory and movement function in AD mice and prevent neurodegeneration [12], [13]. In the present study, a phosphoproteomic profiling of N2aSW cells with or without selenate treatment, was carried to compare the differential phosphoprotein between the N2aSW cells treated with or without sodium selenate and to explore the acting pathways of selenate that possibly involved in the prevention of AD.

In order to avoid missing some differential phosphoproteins, phosphoproteomics of both total proteins and phosphor-enriched proteins from N2aSW cells treated with/without selenate are investigated in the current study. 2-DE gels stained with Pro-Q Diamond and followed by LC-MS/MS analysis were used to identify the altered phosphoproteins. By comprehensive analyses of the two sets of proteomics data, 65 differential phosphoproteins were identified between the selenate-treated and untreated groups, of which 39 were up-regulated and 26 were down-regulated. 203 serine phosphorylation sites, 167 threonine phosphorylation sites and 51 tyrosine phosphorylation sites were identified from these differentially expressed proteins. Bioinformatic analyses revealed that some of the altered phosphoproteins are closely associated with oxidative stress. Others are mainly involved in the metabolic pathways, including proteasome, energy metabolism, cysteine and methionine metabolism, and purine metabolism.

### Oxidative stress

Among these selenate-altered phosphoproteins in N2aSW cells, a subgroup, including elongation factor 2 (EF2), heterogeneous nuclear ribonucleoprotein H (HNRH1), and peroxiredoxins (PRDXs), are regulated by or associated with oxidative stress. Convincing evidences demonstrates oxidative stress as a prominent feature in AD. Oxidative stress can accelerate the progress of AD [20], [21]. In mammalian cells, eukaryotic EF2 catalyses the translocation of peptidyl-tRNA from the A-site to the P-site on the ribosome. The function of EF2 can be regulated by oxidative stress through its phosphorylation level. EF2 is phosphorylated by Ca^2+^/calmodulin-dependent kinase, and its phosphorylation prevents its binding to the ribosome, thus inhibits the translation at the elongation step to slow down protein synthesis and to conserve energy [22]. Garcia et al. reported that EF2 was dephosphorylated significantly in rats after transient cerebral ischemia, and in H_2_O_2_-treated PC12 cells, a cell line derived from nerve growth factor-differentiated pheochromocytoma [23]. Megumi et al. also indicated that the phosphorylated state of EF2 in human SH-SY5Y neuroblastoma cells was altered by oxidative stress [24]. Selenium imposes its biological effect mainly through selenoproteins in vivo, and most of selenoproteins function as antioxidants to maintain redox homeostasis. In the present study, EF2 phosphorylation level was significantly increased in N2aSW cells after selenate-treatment, suggesting that selenate may regulate the phosphorylation level of EF2 through oxidative stress.

Heterogeneous nuclear ribonucleoprotein (HNR, also named hnRNP) is known to be involved in the splicing process and participates in early heat shock-induced splicing arrest. Stone and Collins observed rapid phosphorylation of hnRNP-C1/C1 in response to low concentrations of H_2_O_2_ in human thelial cells [25]. Megumi also found an increase in the phosphorylated form of hnRNP H3 in SH-SY5Y cells after 6-hydroxydopamine-stimulation [24]. H_2_O_2_-stimulated phophorylation of the C-terminal domain of hnRNP by protein kinase CK1α modulated its RNA binding activity. Oxidative stress can induce the phosphorylation of hnRNPs, thus regulates the functions of hnRNPs [26]. In this study, the phosphorylation level of HNRH1 was down-regulated in N2aSW cells after selenate-treatment, indicating that selenate may also impose its effect on the function of HNRH1 via oxidative stress-mediated protein phosphorylation.

PRDXs were used to reduce or splice the oxidized lipid to protect the integrity of cell membrane. Over-expression and gene silence experiments confirmed the function of these proteins in oxidative defense [27]–[29]. Phosphorylation of PRDX6 at Thr-177 can cause a marked increase in phospholipase A(2) activity, thus affects the phosphorylation level of downstream proteins [30]. In the present paper, the phosphorylation levels of PRDXs, including PRDX2, PRDX4, and PRDX6 were altered after selenate treatment, indicating that selenate could maintain cellular redox homeostasis not only by resisting oxidative damage, but also by regulating the phosphorylation of PRDXs.

### Proteasome

The proteasome is composed of 28 individual α-subunits and β-subunits, which are arranged in four rings, with each ring composed of either seven α- or seven β-subunits. The two inner rings of the proteasome are compose of the β-subunits, which possess the proteolytic sits, whereas the α-subunits function to stabilize the proteasome complex. The proteasome can degrade multiple substrates that are important in maintaining neuronal homeostasis, such as damaged and aggregated proteins [31], [32]. NFTs and SPs caused by misfolded and aggregated tau and Aβ, respectively, are pathological hallmarks of AD, which can be degraded by the ubiquitin proteasome system. Increasing evidences show that proteasome plays important roles in AD formation [33]–[35]. Over-expression of mutant APP significantly inhibited proteasome chymotryptic activity in transgenic mice brains and in cultured cortical neurons [36]. In the present study, the total protein levels of some proteasome subunits, including proteasome subunit alpha type (PSA)1, PSA2, proteasome subunit beta type (PSB)2, PSB6 and (26S protease regulatory subunit 6B) PRS6B, are not changed significantly upon selenate treatment (Fold <1.5), however, their phosphorylation levels are increased significantly. The relation between protein phosphorylation and proteasome activity is not fully understood. Jeffery et al. reported that the loss of proteasome activity was not associated with a decrease in proteasome expression and it may be caused by posttranslational modification [34]. Frank et al. reported that a moderate increase of dephosphorylated subunit α7 was found in the 20S proteasome isolated from AD brains [37]. It has been demonstrated that N2aSW cells have elevated extracellular Aβ levels due to the stable transfection of Swedish mutation APP [17]. Our results showed that sodium selenate could decrease extracellular Aβ level and alter the phosphorylation levels of some proteins involved in the proteasome, a pathway for Aβ degradation. Thus, it is reasonable to deduce that the increase of protein phosphorylation may contribute to the activity of proteasome, and selenate ameliorates the pathological process of AD possibly by elevating the phosphorylation of proteasome subunits and thus increasing the activity of proteasome. Further experiments need to be performed to verify the conjecture.

### Energy metabolism

Through KEGG analysis, energy metabolism (i.e., pentose phosphate pathway, pyruvate metabolism and glycolysis/gluconeogenesis) was identified as one of the most significantly altered pathways in N2aSW cells after selenate-treatment (Table 3). Early AD is characterized by a region-specific decline in glucose utilization and by mitochondrial dysfunction, which have deleterious consequences for neurons through increased production of reactive oxygen species (ROS), ATP depletion and activation of cell death processes. Epidemiological, neuropathological and functional neuroimaging evidences implicate global and regional derangements of metabolism and energetics in AD brain [38]–[40]. These reports are consistent with our results in this paper. As summarized in Tables 1 and 2, four proteins in the pentose phosphate pathway (fructose-bisphosphate aldolase A (ALDOA)↑, ribose-phosphate pyrophosphokinase 1 (PRPS1)↑, ribose-5-phosphate isomerase (RPIA)↑ and transaldolase (TALDO)↑), four in the pyruvate metabolism pathway (aldose reductase (ALDR)↓, lactoylglutathione lyase (LGUL)↑, malate dehydrogenase in cytoplasmic (MDHC)↓ and pyruvate kinase isozymes M1/M2 (KPYM)↑), and four in the glycolysis/gluconeogenesis pathway (ALDOA↑, glyceraldehyde-3-phosphate dehydrogenase (G3P)↓, alpha-enolase (ENOA)↑ and KPYM↓) were found to be down- or up-regulated in phosphorylation level (indicated in arrows above) after selenate-treatment, which testify the viewpoint that energy metabolism plays a crucial role in AD pathogenesis.

In adequately oxygenated tissue, the use of glucose usually is glycolysis, which accounts for 10–15% of the glucose metabolized by the brain. Glycolysis not only includes glycolysis itself (metabolism of glucose-6-phosphate to pyruvate), but also the glucose entering the pentose phosphate shunt and glycogen synthesis. Arias et al. indicated that if glycolysis was inhibited, Aβ neurotoxicity was exacerbated in rat hippocampus and in the isolated culture of nerve terminals [41]. Vlassenko et al. confirmed the relationship between AD and those brain systems uniquely reliant on aerobic glycolysis [42]. Vaishnavi et al. reported that the alteration of glycolysis could compensate for the pressure caused by the dysfunction of mitochondrial respiratory chain in TCA cycle [43]. So far, there has been no report on phosphoprotein alteration in this pathway after selenate-treatment. Previous studies demonstrated that both ALDOA and ENOA, two important proteins in this pathway, were altered in phosphorylation level in the hippocampi of AD mice and AD patients [44]. Our results showed that the phosphorylation levels of three proteins, including ALDOA, ENOA and KPYM, were increased after selenate-treatment, while the phosphorylation level of G3PDH, another protein in this pathway, was decreased significantly. These results suggested that sodium selenate may also affect the progress of AD through glycolysis regulation.

Although pyruvate dehydrogenase-involved process is the major pathway of glucose metabolism and a source for energy production, pentose phosphate pathway accounts for a significant fraction of glucose oxidation in the mature central nervous system. The pentose phosphate pathway is a major source of NAPDH, which is a necessary cofactor for the reduction of glutathione disulfide to GSH, a major scavenger of ROS. Thus, the pentose phosphate pathway plays a pivotal role in maintaining cellular redox balance and fighting against oxidative stress [45]–[47]. The fluxes through pentose phosphate pathway was found to be significantly reduced in APP-PS1 transgenic AD mice when compared with the age-matched controls, and this fluxes reduction may lead to a weakened neural defense system in ammonia detoxification and antioxidant reserve, possibly responsible for the compromised neuronal viability and functions in AD [48]. In the present paper, four key signaling proteins (ALDOA, PRPS1, RPIA and TALDO) in the pentose phosphate pathway were up-regulated in phosphorylation level in the selenate-treated N2aSW cells compared with the untreated cells, indicating that selenate may also intervene in the progress of AD via the pentose phosphate pathway. To our knowledge this is the first paper to report protein phosphorylation in this pathway, which provides a new clue for the research in AD pathogenesis.

### Cysteine and methionine metabolism

Cysteine and methionine are two sulfur-containing essential amino acids. Methionine cannot be synthesized by animals, and it is activated by ATP to produce S-adenosylmethionine (SAM). SAM can be used as a methyl group in a series of important transfer reactions to produce S-adenosylhomocysteine (SAH), and SAH hydrolase (SAHH) catalyses the conversion of SAH into Hcy [49]. Cysteine is synthesized from serine through different pathways in different organism groups. In animals, methionine-derived Hcy is used as a sulfur source. Cystathionine-β-synthase (CBS), which is activated by SAM, catalyses the conversion of Hcy to cysathionine, and cysathionine was catalysed by cysathionase to cysteine [50]. Cysteine is metabolized to pyruvate in multiple routes. In the present study, the phosphorylation levels of four proteins in this pathway (ENOPH, S-methyl-5'-thioadenosine phosphorylase (MTAP), SAHH and aspartate aminotransferase in cytoplasmic (AATC)) were altered. ENOPH and MTAP were up-regulated, while SAHH and AATC were down-regulated. It is worthy to note that SAHH was down-regulated in both protein level and phosphorylation level. The decline of SAHH in the selenate-treated N2aSW cells could be a cause of Hcy decrease in the culture medium. Recently, it was reported that Hcy can exacerbate the pathologies of Aβ and tau, and the cognitive deficit in AD mice [51]. Selenate-treatment was found to reduce the Hcy level in culture medium in this (Fig. 5), thus possibly inhibited the pathological features of AD in N2aSW cells through the pathway of cysteine and methionine metabolism.

### Other key phosphoproteins and pathological proteins

Besides the proteins mentioned above, the phosphorylation of some other proteins, such as ubiquitin carboxyl-terminal hydrolase isozyme L1 (UCHL1), vinculin (VINC), and twinfilin (TWF) -2, was also modulated by selenate treatment in N2aSW cells. UCHL1 belongs to the UCH protease family that deubiquitinates ubiquitin-protein conjugates in the ubiquitin-proteasome system (UPS), which plays an important role in cleaning up abnormal proteins. Inhibition of UCHL1 was linked to suppression of apoptosis in certain types of cells [52]–[54]. UCHL1 directly affects the function and location of target proteins, and protects dorsal root ganglions neurons from lipid peroxidation [55]. UCHL1 is also a neuron-specific de-ubiquitinating enzyme, abundantly presented in the brain. Study demonstrated a broad requirement of UCHL1 in the maintenance of the nervous system [56], [57]. It has been reported that UCHL1 S18Y polymorphism is closely related to AD and dysfunction of UPS is also associated with AD [58]. Recessive loss of function of UCHL1 or down-regulation of UCHL1 can lead to early-onset progressive neurodegeneration [59], [60]. However, there has been no paper on the phosphorylation of UCHL1. In the present study the phosphorylation level of UCHL1 was found significantly increased in the N2aSW cells treated with selenate, suggesting that selenate can affect the activity of UPS via modulating the phosphorylation level of UCHL1, thus has an impact on AD formation and progress.

Heat shock protein (HSP)s belong to a family of molecular chaperone proteins. Some of them can repair misfolded proteins and help to transport misfolded proteins to the proteasome [61]. In this study, the phosphorylation levels of HSP7C and HSP90B were altered in N2aSW cells after selenate-treatment. As HSPs are relevant to both oxidative stress and misfolded protein aggregation, the changes of phosphorylation level in HSPs may affect the heat shock response/ubiquitin/proteasomal pathways, resulting in the clearance of Aβ and tau aggregates formed in the pathological process of AD.

VINC is a conserved actin binding protein localized in focal adhesions and cell-cell junctions, where it couples transmembrane proteins with the actin cytoskeleton. VINC phosphorylation has been suggested repeatedly as one of the mechanisms by which focal adhesions mature, and it has even been linked to VINC activation and its recruitment to focal adhesions [62]. In the activated state, VINC binds to α-catenin and this interaction promotes their binding to the actin cytoskeleton to stabilize adhesion junctions [63], [64]. VINC phosphorylation was also found to be increased in PC12 cells treated with nerve growth factor [65]. Phosphorylation of VINC at tyrosine residues 100 and 1065 by Src kinases is a mechanism by which these kinases regulate actin filament assembly and cell spreading [66]. In the present work, 17 serine phosphorylation sites and 16 threonine phosphorylation sites of VINC were found up-regulated in N2aSW cells treated with sodium selenate, suggesting that selenate may regulate actin filament assembly through VINC phosphorylation. As actin filament assembly plays an important role in neurite outgrowth and axon impairment occurs in the early stage of AD, the increase of VINC phosphorylation implicates the potential effect of selenate on neurite formation at the early stage of AD pathogenesis.

TWFs are evolutionarily conserved regulators of cytoskeletal dynamics. Inactivation of the single TWF gene from budding yeast and fruit fly results in defects in endocytosis, cell migration, and organization of the cortical actin filament structures [67], [68]. Drosophila TWF regulates F-actin formation with rapid actin turnover in three different systems: postsynaptic neuromuscular junction (NMJ) synapses, migratory border cells and epithelial follicle cells [67], [69]. RNAi-based screening and overexpression experiments confirmed that TWF2 is a protein involved in neurite outgrowth [70]. Loss of TWF function results in defects in axonal growth in the brain and border cell migration in the ovary [69]. In the present study, total and phophorylated levels of TWF2 were all increased in the N2aSW cells after selenate-treatment, suggesting that selenate may also affect actin formation and thus axon growth by modulating the protein expression and phosphorylation of TWF.

T-complex protein (TCP) family plays a role in the folding of actin and tubulin [71]. Loss of its function can result in aberrantly folded proteins and hence protein aggregation. In this paper, altered phosphorylation level was found in TCPZ, TCPA, and TCPG in N2aSW cells treated with selenate. Those results support the viewpoint that selenate may intervene in the pathological process of AD through TCP phosphorylation.

Based on the proteomic results, some AD pathological molecules, including total and phosphorylated tau, Aβ and its relevant proteins APP and BACE-1, together with Hcy, were selected for biochemical analyses. The extracellular levels of Hcy and Aβ were decreased significantly after selenate-treatment, while the expression levels of APP and BACE1 proteins did not change significantly. The phosphorylation level of tau at pS422 and pS396 were significantly down-regulated in the selenate-treated cells compared to the untreated cells. No significant change was observed in the phosphorylation level of tau at pS404, pT231 and total tau. Previous work by van Eersel et al showed reduction of phosphorylation of tau at multiple sites, while the present study only detected the decreases of phosphorylation of tau at pS422 and pS396. The difference may be caused by the AD models selected. In the study of van Eersel, the AD model mice were used, while in our study AD model cell N2aSW cells were used. The phosphorylated sites of tau can be different in different AD models even though they are treated with the same drug.

Reduced phosphatase activity has been implicated in the formation of hyperphosphorylated tau tangles in AD. A key phosphatase implicated in regulating tau protein phosphorylation is the serine-threonine phosphatase PP2A, which is found colocalised with tau and microtubules in the brains and accounts for more than 70% of tau dephophorylation [72], [73]. The inhibition of PP2A can induce tau hyperphosphorylation and spatial memory deficits, and activation of PP2A is considered as an attractive therapeutic approach in the treatment of AD [74], [75]. Niall et al. reported that sodium selenate acted as a specific agonist for PP2A, significantly boosting phosphatase activity to induce therapeutically relevant dephosphorylation of tau [13]. van Eersel et al also reported that sodium selenate can mitigate tau pathology, neruodegeneration, and functional deficits through the activation of PP2A in AD mice [12]. In the present study, sodium selenate was found to result in a significant decline in tau phosphorylation at pS422 and pS396 in N2aSW cells, indicating that selenate may also implement its effect on AD model cells through the PP2A-mediated pathway. However, further investigation is required to confirm the PP2A-mediated mechanism of selenate on AD.

Collectively, these results indicated that selenate treatment in N2aSW cells can alter the phosphorylation levels of several proteins relating to oxidative stress, metabolic pathways, neurite outgrowth, Hcy alteration, tau phosphorylation, Aβ generation, and protein aggregates clearance. These results provide a comprehensive view for investigating the effect and mechanism of selenate in AD prevention.

## Supporting Information

S1 File **Tables S1 & S2.** Table S1 Differentially phosphorylated peptides identified from the total proteins of selenate-treated and untreated. N2aSW cells. Table S2 Differentially phosphorylated peptides identified from the enriched proteins of the selenate-treated and untreated N2aSW cells.
(DOC)
