# Supplementary material for: Phosphoproteomic Profiling of Selenate-Treated Alzheimer's Disease Model Cells
Source: PLoS One. 2014 Dec 8;9(12):e113307. doi: 10.1371/journal.pone.0113307 (PMC4259334; doi:10.1371/journal.pone.0113307)
Supplement: S1 File — Tables S1 & S2. Table S1 Differentially phosphorylated peptides identified from the total proteins of selenate-treated and untreated. N2aSW cells. Table S2 Differentially phosphorylated peptides identified from the enriched proteins of the selenate-treated and untreated N2aSW cells. (DOC) [file pone.0113307.s001.doc]

Supporting information for

# Phosphoproteomic profiling of selenate-treated Alzheimer's disease model cells

**Ping Chen^1^┴, Lixiang Wang^2^┴, Yong Wang^1^, Shuiming Li^2^, Liming Shen^1^, Qiong Liu^2^*, and Jiazuan Ni^1,2^***

*^1^ College of Life Sciences, Shenzhen Key Laboratory of Microbial Genetic Engineering, Shenzhen University, Shenzhen 518060, China*

*^2^ Shenzhen Key Laboratory of Marine Biotechnology and Ecology, Shenzhen University, Shenzhen 518060, China.*

Supporting Table 1-2

Table S1 Differentially phosphorylated peptides identified from the total proteins of selenate-treated and untreated. N2aSW cells.

| Spot No.^a^ | Protein abbreviation | Protein name^b^ | phosphorylated peptide^c^ |
| --- | --- | --- | --- |
| 1 | UCHL1 | Ubiquitin carboxyl-terminal hydrolase isozyme L1 | | R.FSAVALCK.A  K.QIEELKGQEVSPK.V  K.QFLSETEKLSPEDR.A  K.NEAIQAAHDSVAQEGQCR.V  R.MPFPVNHGASSEDSLLQDAAK.V  R.MPFPVNHGASSEDSLLQDAAK.V | | --- | |
| 2 | TCPA | T-complex protein 1 subunit alpha | | RSLHDALCVVKR  RSQNVMAAASIANIVKS | | --- | |
| 4 | DNJA2 | DnaJ homolog subfamily A member 2 | REGSGGGGGMDDIFSHIFGGGLFGFMGNQSRS |
| 7 | AATC | Aspartate aminotransferase, cytoplasmic | K.IANDNSLNHEYLPILGLAEFR.S |
| 9 | HSP7C | Heat shock cognate 71 kDa protein | K.SFYPEEVSSMVLTK.M |
| 10 | ENOPH | Enolase-phosphatase E1 | K.LLFGHSTEGDILELIDGHFDTK.I |
| 11 | SAHH | Adenosylhomocysteinase | | R.ESLIDGIKR.A  R.GISEETTTGVHNLYK.M  K.DGPLNMILDDGGDLTNLIHTKYPQLLSGIR.G | | --- | |
| 12 | CAZA2 | F-actin-capping protein subunit alpha-2 | | R.TSVETALR.A  K.EATDPRPYEAENAIESWR.T  K.KVDGQQTIIACIESHQFQAK.N  K.IVEAAENEYQTAISENYQTMSDTTFK.A  R.EGAAHAFAQYNLDQFTPVKIEGYEDQVLITEHGDLGNGK.F | | --- | |
| 13 | LGUL | Lactoylglutathione lyase | | R.GFGHIGIAVPDVYSACK.R  K.GLAFIQDPDGYWIEILNPNK.I | | --- | |
| 16 | RTCB | tRNA-splicing ligase RtcB homolog | | K.VFNTTPDDLDLHVIYDVSHNIAK.V  R.TNLDESDVQPVKEQLAQAMFDHIPVGVGSK.G  R.LLRTNLDESDVQPVKEQLAQAMFDHIPVGVGSK.G | | --- | |
| 18 | TCPZ | T-complex protein 1 subunit zeta/T-complex protein 1 subunit zeta-2 | | K.MLVSGAGDIK.L  K.SETDTSLIR.G  K.TEVNSGFFYK.S  K.HKSETDTSLIR.G  R.IITEGFEAAKEK.A  K.VLAQNSGFDLQETLVK.V  K.DGNVLLHEMQIQHPTASiIAK.V  K.VATAQDDITGDGTTSNVLIIGELLK.Q | | --- | |
| 19 | RPIA | Ribose-5-phosphate isomerase | | K.LASHTAVENHVK.N  K.LASHTAVENHVK.N | | --- | |
| 23 | G3PDH | Glyceraldehyde-3-phosphate dehydrogenase | | R.VVDLMAYMASKE.  R.GAAQNIIPASTGAAK.A  R.VPTPNVSVVDLTCR.L  K.LISWYDNEYGYSNR.V  K.IVSNASCTTNCLAPLAK.V  R.VIISAPSADAPMFVMGVNHEK.Y  K.VIHDNFGIVEGLMTTVHAITATQK.T | | --- | |
| 25 | HNRH1 | Heterogeneous nuclear ribonucleoprotein H | | R.GLPWSCSADEVQR.F  R.DLNYCFSGMSDHR.Y  K.HTGPNSPDTANDGFVR.L  K.HTGPNSPDTANDGFVR.L  R.STGEAFVQFASQEIAEK.A  R.STGEAFVQFASQEIAEK.A  R.ATENDIYNFFSPLNPVR.V  R.ATENDIYNFFSPLNPVR.V  R.ATENDIYNFFSPLNPVR.V  R.YGDGGSTFQSTTGHCVHMR.G  R.YGDGGSTFQSTTGHCVHMR.G  R.YGDGGSTFQSTTGHCVHMR.G  R.GLPYRATENDIYNFFSPLNPVR.V  R.GAYGGGYGGYDDYGGYNDGYGFGSDR.F  R.GAYGGGYGGYDDYNGYNDGYGFGSDR.F  R.GAYGGGYGGYDDYNGYNDGYGFGSDR.F  R.RGAYGGGYGGYDDYNGYNDGYGFGSDR.F | | --- | |
| 27 | HYOU1 | Hypoxia up-regulated protein 1 | | R.YFQHLLGK.Q  R.FLGDSAAGMAIK.N  K.LPATEKPVLLSK.D  R.SVQKLEELTLR.D  K.LGNTISSLFGGGTSSDAK.E  R.SRFPEHELIVDPQR.Q  K.LPATEKPVLLSKDIEAK.M  R.LIPEMDQVFTEVEMTTLEK.V  K.TVLSANADHMAQIEGLMDDVDFK.A  K.LYQPEYQEVSTEEQREEISGK.L  R.SLAEDFAEQPIKDAVITVPAFFNQAER.R  K.VIPPAGQTEEAKPILEPDKEETGTEPADSEPLELGGPGAGPEQEEQSAGQK  K.VIPPAGQTEEAKPILEPDKEETGTEPADSEPLELGGPGAGPEQEEQSAGQK | | --- | |
| 28 | HS90B | Heat shock protein HSP 90-beta/Heat shock protein HSP 90-alpha | R.GVVDSEDLPLNISR.E |
| 29 | PRDX4 | Peroxiredoxin-4 | | R.QITLNDLPVGR.S  R.VSVADHSLHLSK.A  R.IPLLSDLNHQISK.D  K.DYGVYLEDSGHTLR.G  K.HGEVCPAGWKPGSETIIPDPAGK.L  R.TRENECHFYAGGQVYPGEASR.V  R.LVQAFQYTDKHGEVCPAGWKPGSETIIPDPAGK.L | | --- | |
| 31 | IDH3A | Isocitrate dehydrogenase [NAD] subunit alpha, mitochondrial | | R.MSDGLFLQK.C  K.TPIAAGHPSMNLLLR.K  K.TPIAAGHPSMNLLLR.K  R.ENTEGEYSGIEHVIVDGVVQSIK.L | | --- | |
| 33 | RLA1 | 60S acidic ribosomal protein P1 | | K.AAGVSVEPFWPGLFAK.A  M.ASVSELACIYSALILHDDEVTVTEDK.I  K.ALANVNIGSLICNVGAGGPAPAAGAAPAGGAAPSTAAAPAEEK.K | | --- | |
| 36 | NDUS1 | NADH-ubiquinone oxidoreductase 75 kDa subunit, mitochondrial | | R.YDHLGDSPK.I  K.AVTEGAQAVEEPSIC.-  R.FASEIAGVDDLGTTGR.G  R.IASQVAALDLGYKPGVEAIR.K  R.IASQVAALDLGYKPGVEAIRK.N | | --- | |
| 37 | RANG | Ran-specific GTPase-activating protein | | R.FASENDLPEWK.E  R.FASENDLPEWKER.G  K.ICANHYITPMMELKPNAGSDR.A  K.ICANHYITPMMELKPNAGSDR.A  K.ICANHYITPM MELKPNAGSDR.A  R.AWVWNTHADFADECPKPELLAIR.F  K.DSHEDHDTSTENADESNHDPQFEPIVSLPEQEIK.T  K.DSHEDHDTSTENADESNHDPQFEPIVSLPEQEIK.T | | --- | |
| 38 | GUAA | GMP synthase [glutamine-hydrolyzing] | | K.TLNMTTSPEEK.R  R.SGNIVAGIANESK.K  K.TLNMTTSPEEK.R  K.TLNMTTSPEEK.R  K.ISQMPVILTPLHFDRDPLQK.Q | | --- | |
| 39 | MDHC | Malate dehydrogenase, cytoplasmic | | K.ENFSCLTR.L  K.GEFITTVQQR.G  K.SAPSIPKENFSCLTR.L  K.VIVVGNPANTNCLTASK.S  K.ELTEEKETAFEFLSSA.  K.NVIIWGNHSSTQYPDVNHAK.V | | --- | |
| 43 | KAP0 | cAMP-dependent protein kinase type I-alpha regulatory subunit | K.VSILESLDKWER.L |
| 47 | SYSC | Serine--tRNA ligase, cytoplasmics | | K.YAGLSTCFR.Q  R.GYTPIYTPFFMR.K  R.GYTPIYTPFFMR.K  R.TICAILENYQAEK.G  K.YLIATSEQPIAALHR.D  R.ELVSCSNCTDYQAR.R  K.EVPLENQLQSMEVTEA.  K.AKEVPLENQLQSMEVTEA.  K.EVMQEVAQLSQFDEELYK.V  R.EIGNLLHPSVPISNDEDADNKVER.I  K.SDDNSYDEKYLIATSEQPIAALHR.D  K.EAVGDDESVPENVLNFDDLTADALAALK.V  K.KEAVGDDESVPENVLNFDDLTADALAALK.V | | --- | |

^a^ Protein IDs were assigned manually. ^b^ Protein names were determined by 2-DE-LC-MS/MS analysis. ^c^ Phosphorylated peptides were identified by MS, in which the phosphorylated amino acids were underlined.

Table S2 Differentially phosphorylated peptides identified from the enriched proteins of the selenate-treated and untreated N2aSW cells.

| Spot No.^a^ | Protein abbreviation | Protein name ^b^ | phosphorylated peptide ^c^ |
| --- | --- | --- | --- |
| 1 | VINC | Vinculin | R.ALASQLQDSLK.D  R.ELTPQVISAAR.I  K.SLLDASEEAIKK.D  R.VGKETVQTTEDQILKR.D  K.CDRVDQLTAQLADLAAR.G |
| 2 | VINC | Vinculin | R.TDAGFTLR.W  R.GQGASPVAMQK.A  R.SLGEIAALTSK.L  K.TISPMVMDAK.A  R.MALLMAEMSR.L  R.ALASQLQDSLK.D  K.MSAEINEIIR.V  R.ELTPQVISAAR.I  K.MTGLVDEAIDTK.S  K.SLLDASEEAIKK.D  K.QVATALQNLQTK.T  R.EVENSEDPKFR.E  K.AQQVSQGLDVLTAK.V  K.AQQVSQGLDVLTAK.V  R.VDQLTAQLADLAAR.G  R.ALASQLQDSLKDLK.A  K.ETVQTTEDQILKR.D  R.VGKETVQTTEDQILKR.D  R.NFTVEKMSAEINEIIR.V  K.CDRVDQLTAQLADLAAR.G  K.LVQAAQMLQSDPYSVPAR.D  K.GWLRDPNASPGDAGEQAIR.Q  K.AIPDLTAPVAAVQAAVSNLVR.V  K.AQMQEAMTQEVSDVFSDTTTPIK.L  K.AQMQEAMTQEVSDVFSDTTTPIK.L  R.VLQLTSWDEDAWASKDTEAMKR.A  K.AQMQEAMTQEVSDVFSDTTTPIK.L  R.TNISDEESEQATEMLVHNAQNLMQSVK.E |
| 3 | EF2 | Elongation factor 2 | K.FSVSPVVR.V  K.SDPVVSYR.E |
|  |  |  | K.GEGQLSAAER.A  M.VNFTVDQIR.A  R.VFSGVVSTGLK.V  K.STLTDSLVCK.A  R.AGETRFTDTR.K  R.NMSVIAHVDHGK.S  R.NMSVIAHVDHGK.S  R.CLYASVLTAQPR.L  R.CLYASVLTAQPR.L  K.KEDLYLKPIQR.T  R.FTDTRKDEQER.C  R.TFCQLILDPIFK.V  R.ETVSEESNVLCLSK.S  K.TGTITTFEHAHNMR.V  R.YLAEKYEWDVAEAR.K  K.AYLPVNESFGFTADLR.S  R.GHVFEESQVAGTPMFVVK.A  R.GHVFEESQVAGTPMFVVK.A  R.GHVFEESQVAGTPMFVVK.A  R.CELLYEGPPDDEAAMGIK.S  K.ARPFPDGLAEDIDKGEVSAR.Q  R.KIWCFGPDGTGPNILTDITK.G  K.YRCELLYEGPPDDEAAMGIK.S  R.IMGPNYTPGKKEDLYLKPIQR.T  R.IMGPNYTPGKKEDLYLKPIQR.T  K.SDPVVSYRETVSEESNVLCLSK.S  R.YVEPIEDVPCGNIVGLVGVDQFLVK.T  R.TILMMGR.Y  K.FSVSPVVR.V  K.SDPVVSYR.E  K.GEGQLSAAER.A  R.VFSGVVSTGLK.V  K.STLTDSLVCK.A  K.STLTDSLVCK.A  R.AGETRFTDTR.K  R.NMSVIAHVDHGK.S  R.NMSVIAHVDHGK.S  R.CLYASVLTAQPR.L  R.FTDTRKDEQER.C  R.TFCQLILDPIFK.V  R.ETVSEESNVLCLSK.S  K.TGTITTFEHAHNMR.V  K.LDSEDKDKEGKPLLK.A  K.AYLPVNESFGFTADLR.S  R.GHVFEESQVAGTPMFVVK.A  K.ARPFPDGLAEDIDKGEVSAR.Q  K.YRCELLYEGPPDDEAAMGIK.S  R.YVEPIEDVPCGNIVGLVGVDQFLVK.T |
| 4 | EF2 | Elongation factor 2 |  |
| 5 | SYK | Lysine--tRNA ligase | K.VTGEDPYPHK.F  R.YLDLILNDFVR.Q  R.LTMFLTDSNNIK.E  K.LPETSLFETEETR.K  K.LPETSLFETEETR.K  K.LPETSLFETEETRK.I  K.EICNAYTELNDPVR.Q  M.ATLQESEVKVDGEQK.L  R.NYKSEEEFVHINNK.L  K.ETAATTETPESTEASPSV.  K.ETAATTETPESTEASPSV.  K.KETAATTETPESTEASPSV.  K.KETAATTETPESTEASPSV.  K.KETAATTETPESTEASPSV.  M.ATLQESEVKVDGEQKLSK.N  K.ITYHPDGPEGQAYEVDFTPPFR.R  K.QLNQTASAPNHTADNGVGAEEETLDPNQYYK.I |
| 6 | HSP7C | Heat shock cognate 71 kDa protein | R.LSKEDIER.M  K.DAGTIAGLNVLR.I  K.GRLSKEDIER.M  K.NSLESYAFNMK.A  K.NSLESYAFNMK.A  R.RFDDAVVQSDMK.H  K.SQIHDIVLVGGSTR.I  R.TTPSYVAFTDTER.L  R.TTPSYVAFTDTER.L  K.SFYPEEVSSMVLTK.M  R.QATKDAGTIAGLNVLR.I  K.SFYPEEVSSMVLTK.M  K.STAGDTHLGGEDFDNR.M  R.IINEPTAAAIAYGLDKK.V  R.IINEPTAAAIAYGLDKK.V  K.LDKSQIHDIVLVGGSTR.I  K.TVTNAVVTVPAYFNDSQR.Q  K.QTQTFTTYSDNQPGVLIQVYEGER.A |
| 7 | TCPG | T-complex protein 1 subunit gamma | K.SMIEISR.T  K.EILSEVER.N  K.ISTPVDVNNR.E  K.TAVETAVLLLR.I  K.KISTPVDVNNR.E  R.GASKEILSEVER.N  R.TLIQNCGASTIR.L  K.AMTGVEQWPYR.A  K.AMTGVEQWPYR.A  K.GISDLAQHYLMR.A  R.EDDVGTGAGLLEIK.K  K.GISDLAQHYLMR.A  R.EMMLSIINSSITTK.V  R.EMMLSIINSSITTK.V  R.IVSRPEELREDDVGTGAGLLEIK.K  R.NVLLDPQLVPGGGASEMAVAHALTEK.S  R.NVLLDPQLVPGGGASEMAVAHALTEK.S  R.NVLLDPQLVPGGGASEMAVAHALTEK.S |
| 8 | DPYL2 | Dihydropyrimidinase-related protein 2 | K.TSPAKQQAPPVR.N  R.NLHQSGFSLSGAQIDDNIPR.R |
| 9 | SYYC | Tyrosine--tRNA ligase, cytoplasmic | K.YLPALGYSK.R  R.TSYYENVIK.A  K.LASAAYPDPSK.Q  R.LSSVVTQHDAK.K  K.KLASAAYPDPSK.Q  K.NSEPEEVIPSR.L  R.TVVSGLVQFVPK.E  K.AMLESIGVPLEK.L  K.AMLESIGVPLEK.L  R.LSSVVTQHDAKK.A  R.VHLMNPMVPGLTGSK.M  R.VHLMNPMVPGLTGSK.M  K.MSSSEEESKIDLLDR.K  K.MSSSEEESKIDLLDRK.E  R.GVDSQGMLLCASVEGVSR.Q  K.MSSSEEESKIDLLDRK.E  K.ILSVEKHPDADSLYVEK.I  R.TVVSGLVQFVPKEELQDR.L  K.VYWGTATTGKPHVAYFVPMSK.I  K.ILSVEKHPDADSLYVEKIDVGEAEPR.T  K.ILSVEKHPDADSLYVEKIDVGEAEPR.T |
| 10 | ATPB | ATP synthase subunit beta, mitochondrial | R.TIAMDGTEGLVR.G  R.TIAMDGTEGLVR.G  K.AHGGYSVFAGVGER.T  R.FTQAGSEVSALLGR.I  K.TVLIMELINNVAK.A  K.VALVYGQMNEPPGAR.A  R.LVLEVAQHLGESTVR.T  K.VLDSGAPIKIPVGPETLGR.I  R.AIAELGIYPAVDPLDSTSR.I  R.AIAELGIYPAVDPLDSTSR.I  R.FLSQPFQVAEVFTGHMGK.L  R.FLSQPFQVAEVFTGHMGK.L  R.FLSQPFQVAEVFTGHMGK.L  R.EGNDLYHEMIESGVINLK.D  R.IPSAVGYQPTLATDMGTMQER.I  R.IPSAVGYQPTLATDMGTMQER.I  R.IPSAVGYQPTLATDMGTMQER.I  R.IPSAVGYQPTLATDMGTMQER.I  K.KGSITSVQAIYVPADDLTDPAPATTFAHLDATTVLSR.A |
| 11 | IF4A1 | Eukaryotic initiation factor 4A-I | R.VLITTDLLAR.G  R.VLITTDLLAR.G  K.GYDVIAQAQSGTGK.T  K.MFVLDEADEMLSR.G  R.KGVAINMVTEEDKR.T  R.KGVAINMVTEEDKR.T  R.GIYAYGFEKPSAIQQR.A |
| 12 | 2-Sep | Septin-2 |  |
| 13 | HNRH1 | Heterogeneous nuclear ribonucleoprotein H | R.GLPWSCSADEVQR.F  R.DLNYCFSGMSDHR.Y |
| 14 | HSP7C | Heat shock cognate 71 kDa protein | R.TTPSYVAFTDTER.L  K.SFYPEEVSSMVLTK.M  K.SFYPEEVSSMVLTK.M  K.STAGDTHLGGEDFDNR.M  R.IINEPTAAAIAYGLDKK.V  K.LDKSQIHDIVLVGGSTR.I  K.TVTNAVVTVPAYFNDSQR.Q |
| 15 | ALDOA | Fructose-bisphosphate aldolase A |  |
| 16 | TWF2 | Twinfilin-2 | K.HLSSCAAPAPLTSAER.E  M.AHQTGIHATEELKEFFAK.A |
| 17 | EEF1-dela | Elongation factor 1-delta | R.GVVQDLQQAISK.L  R.SIQLDGLVWGASK.L  R.ITSLEVENQNLR.G  R.ATAPQTQHVSPMR.Q  R.ATAPQTQHVSPMR.Q  R.ATAPQTQHVSPMR.Q  R.ATAPQTQHVSPMR.Q  R.FYEQMNGPVTSGSR.Q  R.FYEQMNGPVTSGSR.Q  K.DIDLFGSDEEEEDK.E  K.FEEHVQSVDIAAFNK.I  K.DIDLFGSDEEEEDKEAAR.L  K.SLAGSSGPGASSGPGGDHSELIVR.I  K.GATPAEDDEDKDIDLFGSDEEEEDK.E  K.GATPAEDDEDKDIDLFGSDEEEEDKEAAR.L |
| 18 | ALDR | Aldose reductase | K.TTAQVLIR.F  M.ASHLELNNGTK.M  K.TIGVSNFNPLQIER.I  K.YKPAVNQIECHPYLTQEK.L |
| 19 | ENOA | Alpha-enolase | R.SGKYDLDFK.S  R.YITPDQLADLYK.S  K.VNQIGSVTESLQACK.L  R.SGKYDLDFKSPDDPSR.Y  R.SGKYDLDFKSPDDPSR.Y  K.DATNVGDEGGFAPNILENK.E |
| 20 | PRPS1 | Ribose-phosphate pyrophosphokinase 1 |  |
| 21 | PRS6B | 26S protease regulatory subunit 6B |  |
| 23 | 5NT3B | 7-methylguanosine phosphate-specific 5'-nucleotidase | R.CPSSHNILDNSK.I |
|  |  |  |  |
| 24 | PSA1 | Proteasome subunit alpha type-1 | R.ETLPAEQDLTTK.N  K.AQPSQAAEEPAEK.A  R.ALRETLPAEQDLTTK.N |
| 25 | KPYM | Pyruvate kinase isozymes M1/M2 | R.LDIDSAPITAR.N  R.NTGIICTIGPASR.S  K.IISKIENHEGVR.R |
| 26 | MTAP | S-methyl-5'-thioadenosine phosphorylase | K.GTIVTIEGPR.F  K.EHEEAVSVDGVLK.T  R.CHSKGTIVTIEGPR.F  K.YVDTPFGKPSDALILGK.I  K.IGIIGGTGLDDPEILEGR.T  R.TSLRPQTFYDGSHCSAR.G  R.TSLRPQTFYDGSHCSAR.G  R.TWGADVVNMTTVPEVVLAK.E  R.TEKYVDTPFGKPSDALILGK.I  K.IGIIGGTGLDDPEILEGRTEK.Y  K.SLLLTTIPQIGSMEWSETLR.N |
| 27 | ACTBL | Beta-actin-like protein 2 | R.DLTDYLMK.I  R.SYELPDGQVITIGNER.F  R.SYELPDGQVITIGNER.F  R.TTGIVMDSGDGVTHTVPIYEGYALPHAILR.L  R.TTGIVMDSGDGVTHTVPIYEGYALPHAILR.L  R.TTGIVMDSGDGVTHTVPIYEGYALPHAILR.L |
| 28 | CYBP | Calcyclin-binding protein | K.EKPSYDTEADPSEGLMNVLKK.I |
|  |  |  |  |
| 29 | SGTA | Small glutamine-rich tetratricopeptide repeat-containing protein alpha | R.APDRTPPSEEDSAEAER.L |
|  |  |  |  |
| 31 | PRDX6 | Peroxiredoxin 6 |  |
| 32 | PSA2 | Proteasome subunit alpha type-2 | R.YNEDLELEDAIHTAILTLK.E |
|  |  |  |  |
| 34 | GDIR1 | Rho GDP-dissociation inhibitor 1 | K.YKEALLGR.V  K.SIQEIQELDK.D  K.IDKTDYMVGSYGPR.A  R.VAVSADPNVPNVIVTR.L  R.AEEYEFLTPMEEAPK.G  K.SIQEIQELDKDDESLR.K  R.LTLVCSTAPGPLELDLTGDLESFKK.Q  R.LTLVCSTAPGPLELDLTGDLESFKK.Q  M.AEQEPTAEQLAQIAAENEEDEHSVNYKPPAQK.S |
| 35 | EMC8 | ER membrane protein complex subunit 8 | R.SDWTNPEINK.A |
|  |  |  |  |
| 36 | SERB | Phosphoserine phosphatase |  |
| 38 | PSB2 | Proteasome subunit beta type-2 |  |
| 39 | BLVRB | Flavin reductase (NADPH) | R.LQDVTDDHIR.M |
|  |  |  |  |
| 40 | ACTB | Actin, cytoplasmic 1 |  |
| 41 | PSB6 | Proteasome subunit beta type-6 | R.TTTGSYIANR.V  R.TTTGSYIANR.V  R.LAAIQESGVER.Q  R.TTTGSYIANRVTDK.L  R.VTDKLTPIHDHIFCCR.S  R.EGMTKDECLQFTANALALAMER.D |
| 43 | PRDX2 | Peroxiredoxin-2 | K.SLSQNYGVLK.N  R.QITVNDLPVGR.S  R.KEGGLGPLNIPLLADVTK.S  K.SAPDFTATAVVDGAFKEIK.L |
| 47 | KPYM | Pyruvate kinase isozymes M1/M2 | R.LDIDSAPITAR.N  R.NTGIICTIGPASR.S  R.EATESFASDPILYRPVAVALDTK.G |
| 48 | NDKA | Nucleoside diphosphate kinase A | R.NIIHGSDSVK.S  K.FLQASEDLLK.E  K.SCAQNWIYE.-  R.TFIAIKPDGVQR.G  R.VMLGETNPADSKPGTIR.G  K.EISLWFQPEELVEYK.S  M.ANSERTFIAIKPDGVQR.G  K.FLQASEDLLKEHYTDLK.D  K.YMHSGPVVAMVWEGLNVVK.T  K.TGRVMLGETNPADSKPGTIR.G  K.YMHSGPVVAMVWEGLNVVK.T  K.YMHSGPVVAMVWEGLNVVK.T  K.SAEKEISLWFQPEELVEYK.S  K.SAEKEISLWFQPEELVEYK.S |
| 49 | MGDP1 | Magnesium-dependent phosphatase 1 | R.LGVTCIHIR.D  R.LQSLGVPVAAASR.T  R.DGMSLQTLTQGLETFAK.A |
| 50 | MGN | Protein mago nashi homolog | K.SVMEELKR.I |
|  |  |  |  |
| 52 | PFD5 | Prefoldin subunit 5 |  |
| 53 | HINT1 | Histidine triad nucleotide-binding protein 1 | K.AQVAQPGGDTIFGK.I  R.CLAFHDISPQAPTHFLVIPK.K  R.CLAFHDISPQAPTHFLVIPK.K  R.MVVNEGADGGQSVYHIHLHVLGGR.Q  R.MVVNEGADGGQSVYHIHLHVLGGR.Q  K.HISQISVADDDDESLLGHLMIVGK.K  K.KHISQISVADDDDESLLGHLMIVGK.K |
| 54 | LEG1 | Galectin-1 | R.FNAHGDANTIVCNTK.E |
|  |  |  |  |

^a^ Protein IDs were assigned manually. ^b^ Protein names were determined by 2-DE-LC-MS/MS analysis. ^c^ Phosphorylated peptides were identified by MS, in which the phosphorylated amino acids were underlined.
